# 2D Scanning Micromirror with Large Scan Angle and Monolithically Integrated Angle Sensors Based on Piezoelectric Thin Film Aluminum Nitride

**DOI:** 10.3390/s20226599

**Published:** 2020-11-18

**Authors:** Katja Meinel, Marcel Melzer, Chris Stoeckel, Alexey Shaporin, Roman Forke, Sven Zimmermann, Karla Hiller, Thomas Otto, Harald Kuhn

**Affiliations:** 1 Center for Microtechnologies, Chemnitz University of Technology, 09111 Chemnitz, Germany; marcel.melzer@zfm.tu-chemnitz.de (M.M.); chris.stoeckel@zfm.tu-chemnitz.de (C.S.); sven.zimmermann@zfm.tu-chemnitz.de (S.Z.); karla.hiller@zfm.tu-chemnitz.de (K.H.); thomas.otto@enas.fraunhofer.de (T.O.); harald.kuhn@enas.fraunhofer.de (H.K.); 2 Fraunhofer Institute for Electronic Nano Systems ENAS, 09126 Chemnitz, Germany; alexey.shaporin@enas.fraunhofer.de (A.S.); roman.forke@enas.fraunhofer.de (R.F.)

**Keywords:** 2D, AlN, aluminum nitride, angle sensor, micromirror, microscanner, two-dimensional scanning, piezoelectric

## Abstract

A 2D scanning micromirror with piezoelectric thin film aluminum nitride (AlN), separately used as actuator and sensor material, is presented. For endoscopic applications, such as fluorescence microscopy, the devices have a mirror plate diameter of 0.7 mm with a 4 mm^2^ chip footprint. After an initial design optimization procedure, two micromirror designs were realized. Different spring parameters for x- and y-tilt were chosen to generate spiral (Design 1) or Lissajous (Design 2) scan patterns. An additional layout, with integrated tilt angle sensors, was introduced (Design 1-S) to enable a closed-loop control. The micromirror devices were monolithically fabricated in 150 mm silicon-on-insulator (SOI) technology. Si (111) was used as the device silicon layer to support a high C-axis oriented growth of AlN. The fabricated micromirror devices were characterized in terms of their scanning and sensor characteristics in air. A scan angle of 91.2° was reached for Design 1 at 13 834 Hz and 50 V. For Design 2 a scan angle of 92.4° at 12 060 Hz, and 123.9° at 13 145 Hz, was reached at 50 V for the x- and y-axis, respectively. The desired 2D scan patterns were successfully generated. A sensor angle sensitivity of 1.9 pC/° was achieved.

## 1. Introduction

Micromirrors are highly functional, miniaturized systems that combine semiconductor technology and optics. As one or two-dimensional deflection units, they are a prerequisite of numerous photonic applications. The range of applications extends from consumer electronics, like laser projection systems, to medical applications, like fluorescence microscopy. Every application has certain requirements and restrictions regarding precision, fabrication limitations, dynamic range, and miniaturization. In the literature, micromirrors are mainly classified based on their excitation principles. These are electrostatic, electrothermal, electromagnetic, and piezoelectric micromirrors [1,2,3]. The latter principle is becoming more and more preferable due to its advantages of a high degree of miniaturization, the possibility for a monolithic integration of actuators and sensor elements, moderate excitation voltages, and high dynamic ranges.

For micromirrors, resonant operation is usually essential for achieving large tilt angles. Variations of the ambient conditions, such as temperature change or external mechanical vibrations, lead to resonance frequency deviations and thus to a change of the tilt angle. Finally, this results in errors in image formation and reconstruction [1,4]. A closed-loop control, which requires position sensors, increases the precision for resonant scanning. Piezoelectric micromirrors offer the advantage of a monolithic integration of sensors and actuators without additional process steps. Thus, there is no need for a heterogeneous integration of additional components, such as external electrodes, photodetectors, coils, or an additional power supply [1,3,4,5].

In the literature, piezoelectric scanners are mainly based on lead zirconate titanate (PZT) due to its high piezoelectric coefficients, which means high deflections at low voltages. Aluminum nitride (AlN) is an alternative piezoelectric transducer material for excitation, as well as for sensor applications. It is not a ferroelectric material, so it has no Curie temperature, or material hysteresis, and shows no aging effects [6,7]. Furthermore, AlN is compatible with standard micro-electro-mechanical systems (MEMS) processes. Since the first publication in 2018 by Shao et al. [6] the number of publications on AlN-based micromirrors has increased significantly. Shao et al. published a micromirror with a 0.2 × 0.2 mm^2^ mirror plate area. The mirror plate is deflected using two L-shaped bending actuators with AlN as the transducer material. Due to the small d_31_-coefficient of AlN, a maximum scan angle of 4°, at 5 V and 63.3 kHz resonant operation, is achieved. In our previous work in 2019 [8], resonant AlN micromirrors with a large scan angle of 104.9° at 20 V and 1.9 kHz were realized for the first time, using an optimized lever design. Other publications on a 1D mirror with integrated sensors followed in 2019 and 2020 [9,10]. With a scan angle of 137.9° at 20 V, 3.4 kHz and a 2 × 3 mm^2^ footprint, these are the AlN scanners with the largest scan angle based on our current knowledge. In October 2019, Pensala et al. [11] proposed a wobbling mode AlN-based LIDAR scanner for automotive applications. A scan angle of 30° at 1 V was reached at 1.6 kHz by the micromirror with a 6.75 × 6.75 × 2 mm³ chip size and 4 mm aperture. At the beginning of 2020, Senger et al. [12] presented an AlN-based Lissajous scanner for smart headlights with a scan pattern of 50° × 20°. For the microscanners of Pensala et al. and Senger et al. a vacuum package is required to realize high scan angles. The micromirrors presented so far are exclusively resonant-operated scanners, to achieve a sufficiently high tilt angle. At the beginning of 2019, Gu-Stoppel et al. published a lever principle to implement a quasi-static scanner [13]. The mirror plate is mounted onto a pillar, which is deflected by four actuators based on aluminum scandium nitride (AlScN). This lever arrangement achieves a high static scan angle of 50° at 150 V_DC_. The challenges with this concept are the high excitation voltage, a complex manufacturing process, and the joining of three wafers using different wafer bonding processes.

In the present paper, resonant driven 2D scanning micromirrors enhanced with a piezoelectric thin film aluminum nitride as a transducer material for actuation and sensing are proposed. Design approach, fabrication, and characterization procedure are described in detail. Possible applications are consumer electronics, such as laser projection scanners. Due to their small footprint, the devices are also suitable for endoscopic applications such as in vivo fluorescence microscopy. An iterative ANSYS FEM simulation was carried out in order to achieve the targeted resonance frequencies. In addition, the influence of layer stresses on the eigenfrequency was analyzed. In comparison to the previous work [8,9,10], doped Si (111) was used both as a bottom electrode and as the device silicon layer to support a high C-axis oriented growth of AlN. The fabricated micromirror devices were characterized and compared in terms of their scanning and sensor characteristics in air.

## 2. Design and Modeling

### 2.1. Design

**Figure 1:**
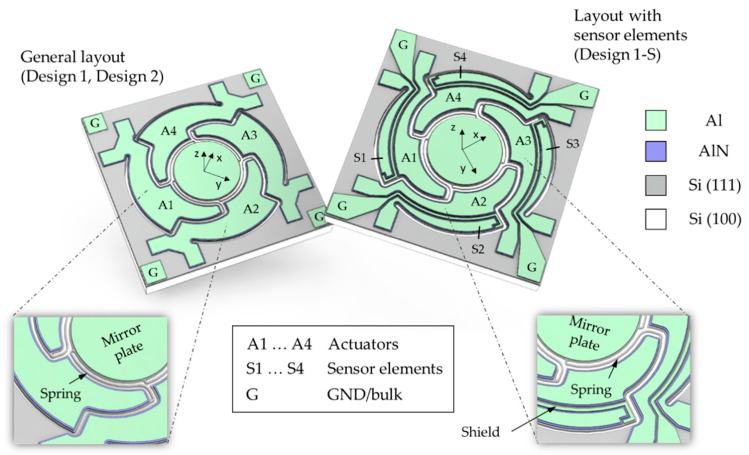
Schematic of the presented aluminum nitride (AlN) micromirror designs.

In Figure 1 a schematic of the micromirror designs is shown. In general, a round mirror plate is connected by S-shaped spring elements with four AlN actuators (A1 … A4). In order to be suitable for endoscopic applications, the micromirror devices were designed with a chip size of 2 × 2 mm^2^, with a mirror plate diameter of 0.7 mm. Two layouts with different spring parameters were realized to achieve spiral (Design 1) or Lissajous (Design 2) scan patterns. To generate spiral patterns, the four actuators are excited with a phase shift of 90° at the same frequency [14]. So, the four spring elements of Design 1 have the same spring widths of 10 µm. However, to generate Lissajous scan patterns, two axes are excited independently with different frequencies. [14,15,16]. To realize a tilt movement around the x-axis, the actuators A2 and A4, and around the y-axis the actuators A1 and A3 are excited with a 180° phase shift to each other. For Design 2, the spring elements for x- and y-axis are dimensioned with different spring widths of 7.5 µm and 10 µm, respectively, to obtain the different resonance frequencies with a sufficient frequency separation. To enable a closed-loop control, an additional layout with sensor elements (S1 … S4) at the actuator edges was introduced (Design 1-S). To avoid cross-coupling, a 20 µm wide shield electrode with ground potential (GND/G) was placed between the sensor element and the actuator. An overview of the designed spring parameters of the micromirror designs is given in Table 1.

The target eigenfrequency of the tilt modes was chosen so that the scanner is mechanically stable and fast enough for high image frame rates. However, a higher stiffness, and thus high eigenfrequency, resulted in a reduction of the deflection [17]. So, in order to realize a mechanically stable system with high deflections, a finite elements (FE) design optimization was necessary to find a compromise between these opposing specifications. The eigenfrequency of the micromirrors is mainly determined by the spring parameters. In order to have mechanically stable springs, the relatively high spring widths of 7.5 µm and 10 µm were chosen. However, to keep the eigenfrequency low, the spring elements were arranged between the actuators and mirror plate to realize long springs despite the small space. This spring layout also reduces intrinsic stress, induced by the aluminum and aluminum nitride layers.

### 2.2. FE Simulation

**Figure 2:**
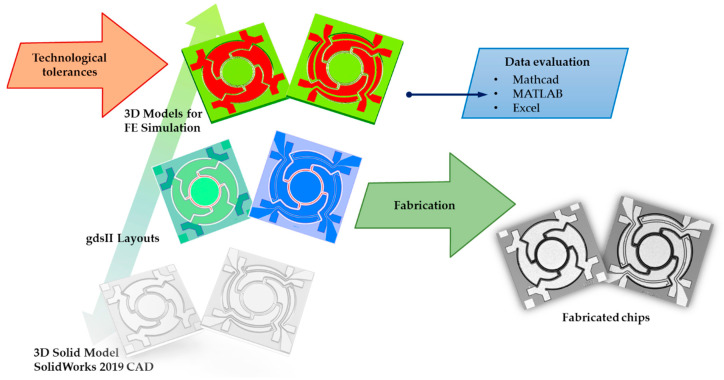
The design procedure.

The simulations were mostly performed in ANSYS. Additional tools were LinkCad, for CAD data translation between different formats, and Matlab, Mathcad, and Excel for data analysis. The FE models were mask conformed without any simplifications, and all layers were taken into account. A schematic of the design procedure is shown in Figure 2. Since a free shape MEMS design was used, the design procedure started in a SolidWorks CAD. Subsequently, the created parametrical free shape micromirror models were converted to an ANSYS FEM simulation environment. Modal and static analysis were carried out to determine the micromirrors key parameters. Various shapes and spring configurations were tested to find the design best fitting the desired specifications. Finally, for selected configurations, layouts in gdsII format were generated for device fabrication.

The material properties for Si (111) are well known from the literature [18,19,20,21,22,23]. The mechanical properties of Al, and especially AlN, were the subject of a detailed investigation. In this paper, values that match our previous parameter identification experiments were used. Table 2 summarizes the parameters used for the micromirror design and modeling.

**Figure 3:**
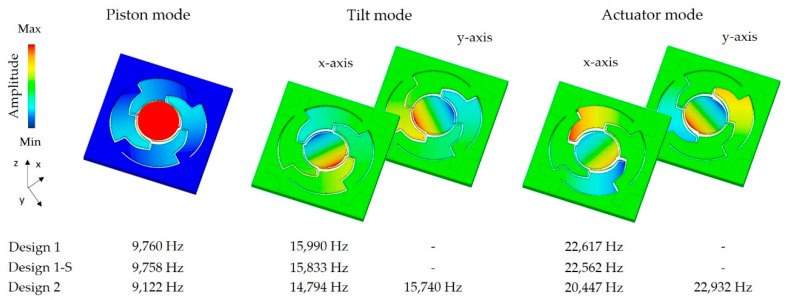
Resulting out-of-plane modes up to 25,000 Hz for both micromirror designs simulated with ANSYS FEM with regard to the parameters summarized in Table 2.

In Figure 3 the modal analysis results for the target geometry of Design 1 and Design 2 are shown. Both designs have the same resonance mode order. First a piston mode appears, resulting in a mirror plate displacement in the z-axis. After the piston mode, the targeted tilt modes for the rotation appear, followed by actuator modes. For Design 2, tilt modes and actuator modes appear separately according to the x- and y-axis. Due to a numerical effect, there is a minimal difference in the eigenfrequency of the x- and y-axis movement for tilt and actuator mode of Design 1. In the FE simulation, these eigenfrequencies could be considered the same. The slightly different structured aluminum layers of Design 1 and Design 1-S result in marginal differences in the eigenfrequencies for these designs.

**Figure 4:**
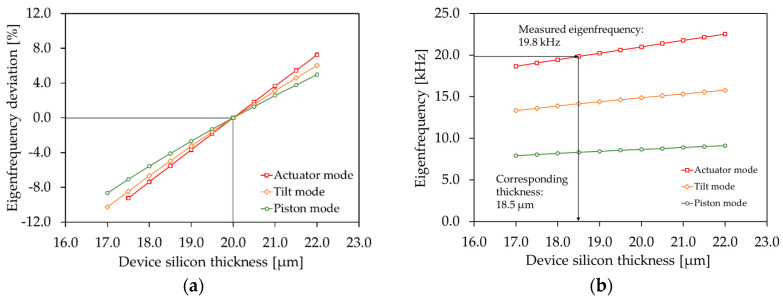
Simulated influence of the device silicon thickness on the eigenfrequency (Design 1): (**a**) eigenfrequency deviation (deviation with respect to the eigenfrequency at 20 µm) versus device silicon thickness; (**b**) eigenfrequency versus device silicon thickness.

Due to technological deviations like underetching, variations of the device silicon layer, and intrinsic prestresses, the measured resonance frequencies differed considerably from the simulated targeted eigenfrequencies, shown in Figure 3. Thus, the FE model was adapted taking into account the measured values (shown in Section 5). The underetching was measured as 0.75 µm at each side of the springs (1.5 µm cumulative), resulting in effective spring widths of 8.5 µm and 6.0 µm for the designed 10 µm and 7.5 µm spring widths (see Section 3). In Figure 4a the simulated eigenfrequency deviation (deviation with respect to the eigenfrequency at 20 µm) of the piston, tilt, and actuator mode versus the device silicon thickness is depicted for Design 1. The eigenfrequency versus the device silicon thickness is shown in Figure 4b. In comparison to the piston and tilt mode, the eigenfrequency of the actuator mode is mostly dependent on the device silicon thickness. Subsequently, in order to determine the effective silicon thickness, the measured resonance frequency of the actuator mode was compared with the calculated values. An effective device silicon thickness of 18.5 µm was determined (see Figure 4b).

**Figure 5:**
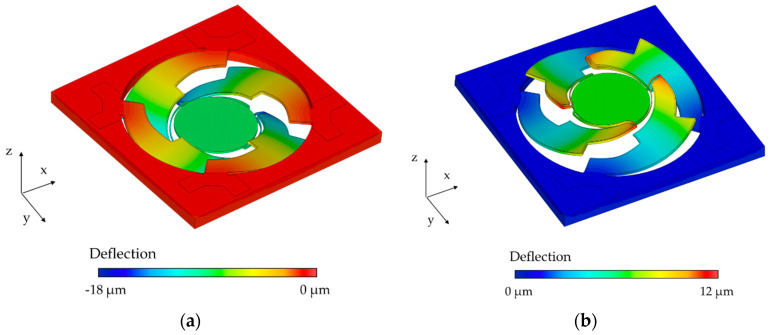
Simulated influence of intrinsic prestress on the actuator offset-deflection: (**a**) tensile stress at wafer center; (**b**) compressive stress at wafer edge.

Furthermore, the influence of intrinsic stress on the eigenfrequency had to be evaluated. The bending actuators could be considered as a layer stack of silicon, aluminum nitride, and aluminum. Different material properties and temperature changes during the fabrication process led to a prestressed state (up to several hundred MPa) in the micromirrors after fabrication. Both positive (tensile stress, actuators offset-deflection in negative z-direction) and negative effective stresses (compressive stress, actuators offset-deflection in positive z-direction) were observed in the fabricated microsystems, varying across the wafer. The actuator deflections, to determine the intrinsic stress by a prestressed static analysis, were measured in the wafer center and wafer edge by white light interferometry (WLI) (see Section 5.1). Figure 5 shows the simulation results. An actuator deflection in both negative (wafer center) and positive (wafer edge) z-direction becomes evident. The absolute values of the effective stress, corresponding to the measured actuator deflections, is approximately 350 MPa and −250 MPa for Figure 5a,b, respectively.

**Figure 6:**
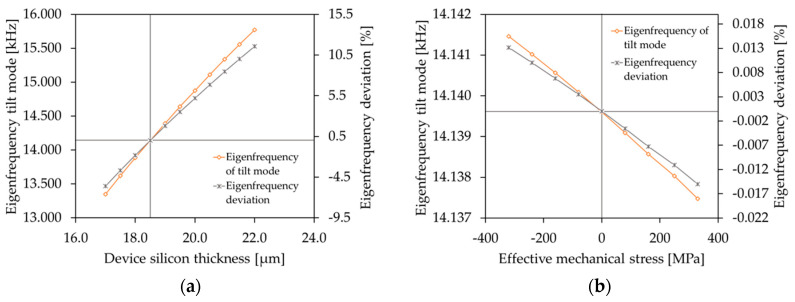
Comparison of the simulated influences on the eigenfrequency of the tilt mode (Design 1): (**a**) eigenfrequency and eigenfrequency deviation (deviation with respect to the eigenfrequency at 18.5 µm) versus device silicon thickness; (**b**) eigenfrequency and eigenfrequency deviation (deviation with respect to the zero stress level) versus the effective mechanical stress.

To investigate the influences of both the tensile effective stresses and the compressive effective stresses on the eigenfrequency of the tilt mode, a prestressed modal analysis was carried out. In Figure 6 the influences of deviations of the device silicon thickness and the intrinsic stresses on the eigenfrequency of the tilt mode are compared. The eigenfrequency and eigenfrequency deviation (deviation with respect to the eigenfrequency at 18.5 µm) versus the device silicon thickness is depicted in Figure 6a. However, in Figure 6b the eigenfrequency and eigenfrequency deviation (deviation with respect to the zero stress level) versus the effective mechanical stress is shown. The stress level range is ± 400 MPa. Even high stress levels resulting in a significant deflection of the actuators led to small changes of the eigenfrequency of the tilt mode (as low as 0.02% or 3 Hz). Thus, the influence of the varying silicon thickness was more dominant than intrinsic stress. The simulation results were highly comparable with the quantitative and qualitative results of the white light interferometer measurement in Section 5.1.

Additional technological tolerances, such as defects in the AlN thin film or notching effects on the spring backsides or the whole diaphragm, which increase the effective size of the actuators, were not taken into account in the presented model.

## 3. Fabrication

**Figure 7:**
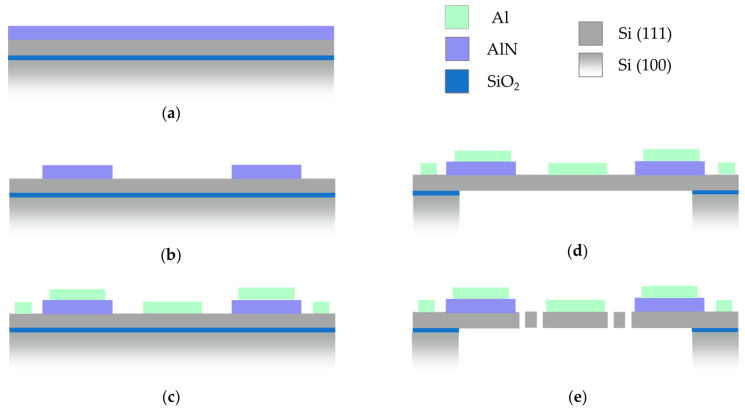
The fabrication process flow: (**a**) AlN deposition; (**b**) AlN wet etching; (**c**) Al deposition and structuring; (**d**) Backside Si etching by deep reactive ion etching (DRIE); (**e**) Device Si structuring.

The overall process flow is depicted in Figure 7. To realize the targeted MEMS, 150 mm bonded silicon-on-insulator (BSOI), wafers with a Si (100) bulk substrate and a Si (111) device silicon layer with a conductivity of <0.1 Ωcm and a thickness of 20 µm were used as base material. The buried oxide thickness was 1 µm. Before the deposition of the piezoelectric AlN layer, the wafers were cleaned by means of an RCA-clean. Furthermore, the native oxide on the wafer surface was removed by an HF dip. Subsequently, 600 nm c-axis oriented AlN was deposited using DC magnetron sputtering [24] (see Figure 7a). The layers were deposited using a pulsed double ring DC magnetron (DRM 250). The inner target had a diameter of 120 mm and an on-off duty cycle of 100/2 µs. A power of 220 W was applied to this target. The outer ring-shaped target had an inner diameter of 123 mm and an outer diameter of 236 mm. The duty cycle was 95/2 µs with an applied power of 1900 W. Both targets were pure Al targets (5N5). The AlN deposition was performed under pure nitrogen atmosphere (50 sccm) at a pressure of 0.7 Pa. The heating power of the wafer heater was adjusted to a targeted temperature of 350 °C. At the used target-wafer distance of 75 mm, a deposition rate of approx. 0.5 nm/s was achieved. In [25], the crystalline quality of the sputtered AlN films on Si (111) was characterized.

**Figure 8:**
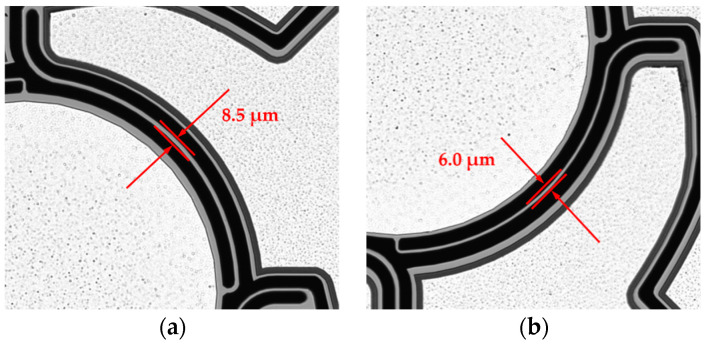
Microscope image of the spring structures of a fabricated micromirror of Design 2. Technological deviations led to smaller resulting spring widths: (**a**) The 10 µm springs have a resulting width of 8.5 µm (**b**) The 7.5 µm springs have a resulting width of 6.0 µm.

**Figure 9:**
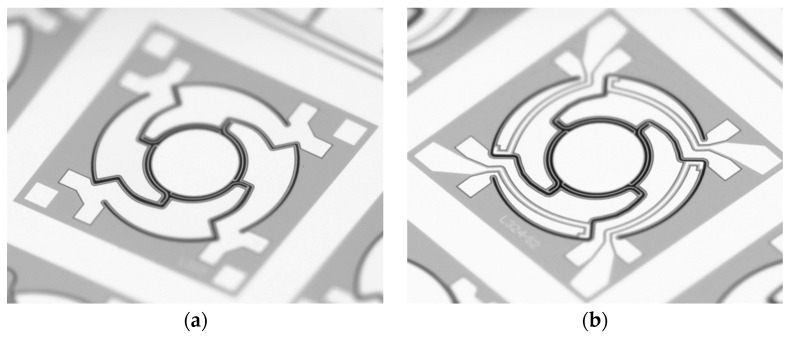
Photography of the processed micromirror devices on wafer level captured by a stereo microscope: (**a**) Design 2; (**b**) Design 1-S.

**Figure 10:**
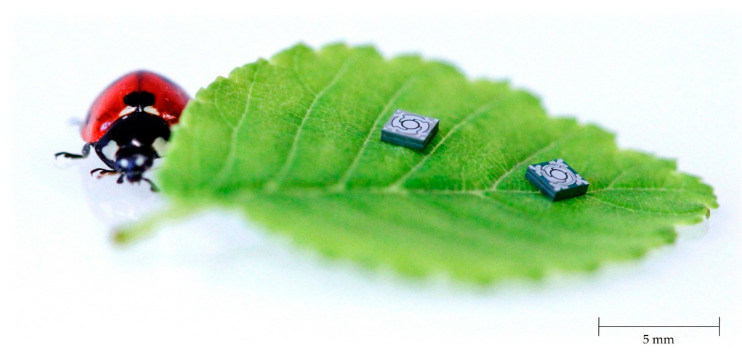
Diced micromirrors of Design 2 (left) and Design 1-S (right) in size comparison to a ladybug (Coccinella septempunctata).

Following the AlN deposition, the AlN layer was wet-chemically patterned in phosphoric acid (85% phosphoric acid at 80 °C) using a SiO_2_ hard mask (see Figure 7b). In the following steps aluminum was deposited and structured, which served as upper electrode as well as contact to the Si substrate (bottom electrode) (see Figure 7c). Subsequently, in Figure 7d, the 550 µm thick Si-handle layer on the backside of the wafer was structured down to the 1 µm thick buried oxide, which served as an etch stop. This buried oxide was then removed by dry etching. In Figure 7e, the 20 µm Si-device layer was patterned to release the moving structures. During this process, the backside of the wafer was covered with an approximately 8 µm thick spray resist. Under the selected process conditions, there was a slight undercutting of the resist mask. Therefore, the final spring elements were narrower compared to the layout (see Figure 8). Finally, the photoresist was removed by plasma paint removal (PLE). Figure 9 shows two manufactured chips. A photography of diced micromirrors of Design 2 and Design 1-S, in size comparison with a ladybug, is shown in Figure 10.

## 4. Experimental Setup

### 4.1. Optical Measurements

The topographical area scans to identify the influence of layer stresses in Section 5.1 were carried out with a white light interferometer (WLI) (Zygo NewView 6300). In order to achieve a maximum image resolution, the micromirror frame was cut to a narrow strip in the WLI software.

The deflection measurements in Section 5.2 and Section 5.4 were performed by laser-Doppler-vibrometry (LDV). To pre-characterize the micromirrors, motion scans were performed by a Polytec MSA 400 laser-Doppler-vibrometer with an OFV 5000 controller to identify the individual resonance modes. For actuation, a sinusoidal chirp signal with 1 V amplitude was applied. For a statistical determination of the resonance frequency and the associated amplitude, frequency response curves were recorded over the entire wafer. For this measurement an automatic probe bench (Cascade Microtech PA 200) was used in combination with a Polytech LDV with an OFV 3001 controller. Due to the measuring device, only one actuator was excited at a time. The frequency response curve was recorded at the mirror plate edge towards the corresponding actuator. The amplitude *δ* and the resonance frequency of the individual modes were calculated using a script written in C# (Visual Studio 2019 development environment). With the calculated deflection and the mirror diameter *b* = 700 µm, the resonant mechanical tilt angle *ϕ_mech_* could be determined with (1).

*ϕ_mech_* = sin^−1^(*δ* / 0.5 ∙ *b*). (1)

### 4.2. High-Deflection Measurements

**Figure 11:**
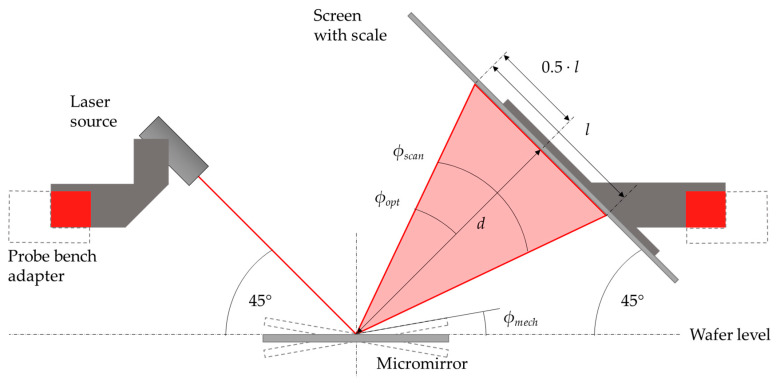
Schematic of the optical test setup. The laser beam and screen were aligned in a defined distance at a 45 ° angle to the mirror. The illustrated scan angle, optical angle, and mechanical tilt angle could be calculated with (2) and (3).

**Figure 12:**
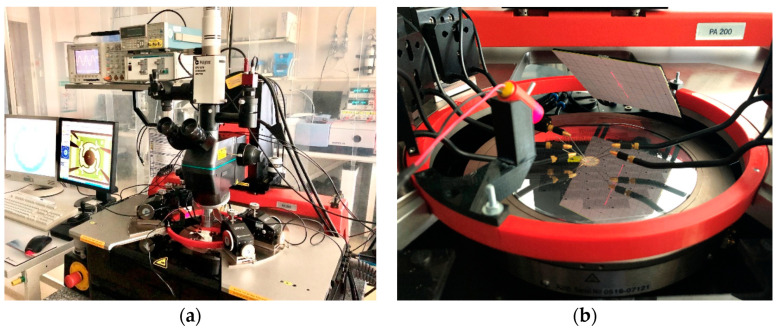
Photograph of the optical test setup: (**a**) Entire setup around the automated probe bench station; (**b**) Integrated probe bench adapter with laser source holder and scaled screen.

The previously mentioned laser-Doppler-vibrometer is not suitable for mechanical tilt angles above 10° degrees, since the laser beam deflected by the mirror is no longer completely translated into the device objective lens. In the previous work [10] a high deflection setup was realized to characterize the micromirrors, with regard to their scan characteristics for tilt angles up to 36°. The construction of the 3D printed setup was adapted for installation in the mentioned probe station to realize a quick-mount, optical characterization of the microsystem, with defined angles and distances. A laser source (660 nm, laser class 1) and a screen were fixed at a 45° angle to the wafer. The laser source projected a laser beam onto the mirror surface, where the beam was reflected on the screen. The optical angle *ϕ_opt_* could be calculated with the measured length 0.5 · *l* of the scan line, and the defined distance *d* between mirror and screen (1). The mechanical tilt angle *ϕ_mech_* and the optical scan angle *ϕ_scan_* are defined with (2). The measurements to determine the scanning behavior at high deflections and high voltages in Section 5.2 were carried out using a Tektronix AFG 320 function generator, with a TEGAM 2350 high-voltage amplifier. Since the high-voltage amplifier only has two analog output channels, only two actuators (one axis) were excited with 180° phase shift at a time. Figure 11 depicts a schematic of the measurement setup. In Figure 12a a photograph of the entire setup, mounted to the automatic probe station, and in Figure 12b the high deflection setup is shown.

*ϕ_opt_* = tan^−1^(0.5 · *l*/*d*), (2)

*ϕ_scan_* = 2 · *ϕ_opt_* = 4 · *ϕ_mech_*. (3)

### 4.3. Pattern Generation

The scan pattern evaluation was carried out in combination with the optical measurement setup from Section 4.2. A multifunction input/output (I/O) device from National Instruments (NI USB 6363 BNC) was used as signal generator. In conjunction with LabVIEW for programming, the micromirrors were excited. The four actuators were connected with the analog output channels of the I/O device (AO1 … AO3). To obtain a Lissajous scan pattern, the actuators associated with the x-axis (A2, A4) and the y-axis (A1, A3) were excited orthogonally with two sine functions, *x(t)* and *y(t)* for the scan paths in the x- and y-direction (4) [14,15,16]. *A_x_* and *A_y_* represent the maximum scan amplitudes, and *φ_x_* and *φ_y_* the scan phases for the x- and y-direction, respectively. *t* is the time-variable. The scan frequencies, *f_x_* and *f_y_*, correspond to the resonance frequency of the tilt modes for the x- and y-axis.

*x(t)* = *A_x_* ∙ sin(2π*f_x_* ∙ *t* + *φ_x_*),     *y(t)* = *A_y_* ∙ sin(2π*f_y_* ∙ *t* + *φ_y_*). (4)

To realize a spiral scan pattern, the four actuators were actuated with the two waveforms *x(t)* (A2 and A4) and *y(t)* (A1 and A3), with the same amplitude, *A_x_* = *A_y_*, and frequency, *f_x_* = *f_y_*, and with a phase shift of *Δφ* = 90° to each other [14]. This frequency corresponds to the resonance frequency of the tilt mode. In order to obtain a filled pattern, the amplitude was modulated with a triangular signal. Due to technological deviations, the amplitude and resonance frequency of the four actuators differed, so that the amplitude of the several actuators had to be adjusted to get a circle. Table 3 summarizes the parameters for actuator excitation to generate the corresponding scan patterns.

### 4.4. Sensor Characterization

**Figure 13:**
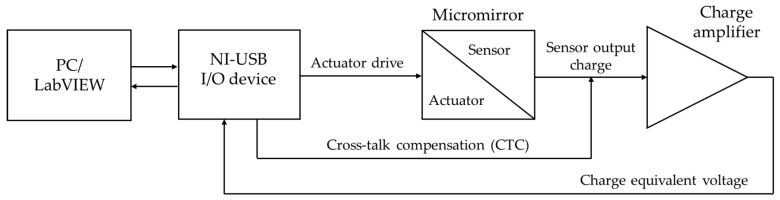
Schematic of the sensor signal evaluation setup [10].

**Figure 14:**
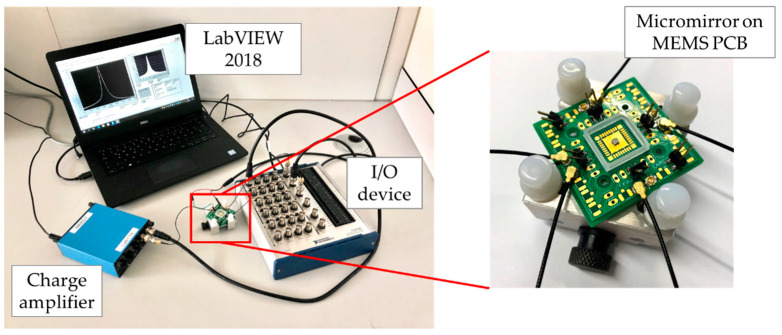
Photograph of the sensor characterization setup.

A schematic and a photograph of the sensor signal evaluation setup are given in Figure 13 and Figure 14. The multifunction I/O device mentioned in Section 4.3 was used as signal generator for the micromirror drive. LabVIEW was used for the control of the micromirror drive signal and for later data acquisition. For the actuator excitation, a sinusoidal sweep signal was used to obtain the desired tilt movement. The generated sensor charge signal was amplified and converted to an analog charge-equivalent voltage by a commercial charge amplifier (MMF M68D1). A gain factor of 1000 mV/pC was chosen for a high signal-to-noise ratio. For digitization, the analog voltage signal of the charge amplifier was recorded by the I/O device. The data evaluation was synchronized with the excitation. The converted and recorded sensor signal was digitally filtered, and a frequency response curve was generated by performing a fast Fourier transformation. The charge at the resonance frequency was determined from this curve by an algorithm. Due to cross-talk between the sensor and actuator, which led to an incorrect charge determination, a cross-talk compensation (CTC) was applied. This compensation consisted of a capacitance-coupled voltage signal, which was proportional and 180° phase-shifted to the actuator drive voltage. A further description of the sensor characterization setup and CTC was given in the previous work [10].

## 5. Results and Discussion

### 5.1. White Light Interferometry

**Figure 15:**
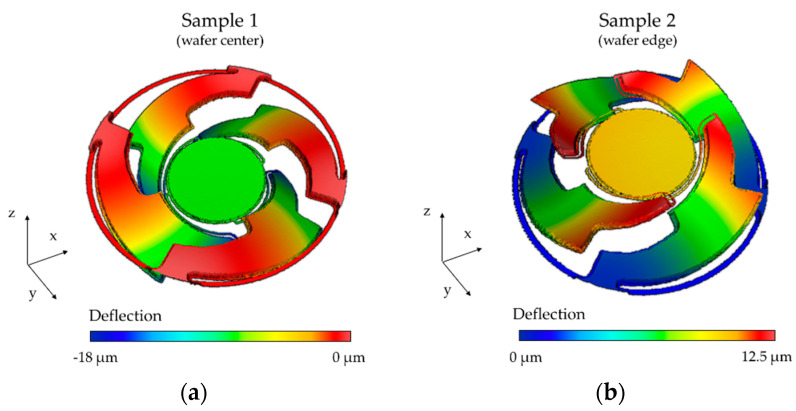
Topological area scans of Design 1 performed using white light interferometry: (**a**) wafer center (Sample 1); (**b**) wafer edge (Sample 2).

**Figure 16:**
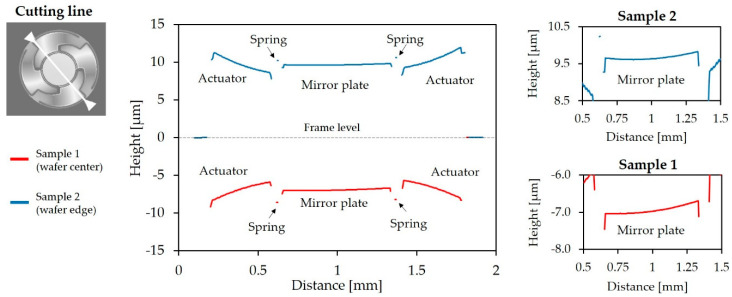
Cross-section of the sample micromirrors (Design 1) of the wafer center (Sample 1) and the wafer edge (Sample 2). A zoomed section of the corresponding mirror plate is shown on the right.

Topological area scans of a mirror sample of the wafer center and the wafer edge are depicted in Figure 15a,b, respectively. The actuators of the micromirrors at the wafer center show an offset deflection in a negative z-direction, while the actuators at the wafer edge show an offset-deflection in positive z-direction, induced by tensile and compressive stresses of the actuator layers. Cross-sections of the sample micromirrors at the wafer center, and at the wafer edge, are given in Figure 16. Compared to the stress-deflected actuators, the mirrors showed a relatively low offset deflection (resulting in a 0.029° and 0.015° offset tilt angle for Sample 1 and Sample 2) due to the spring design and the symmetrical actuator arrangement. A curvature of the mirror plates, induced by the reflective aluminum layer, also became apparent. A mean static curvature of 107 nm and a radius of curvature of 0.5 m were calculated. Future work will take into account the reduction of the curvature by increasing the device Si layer thickness. In addition, the stress induced by the front side aluminum layer is planned to be compensated for by the deposition of an aluminum layer of the same thickness on the backside of the mirror plate.

### 5.2. LDV Measurements

**Figure 17:**
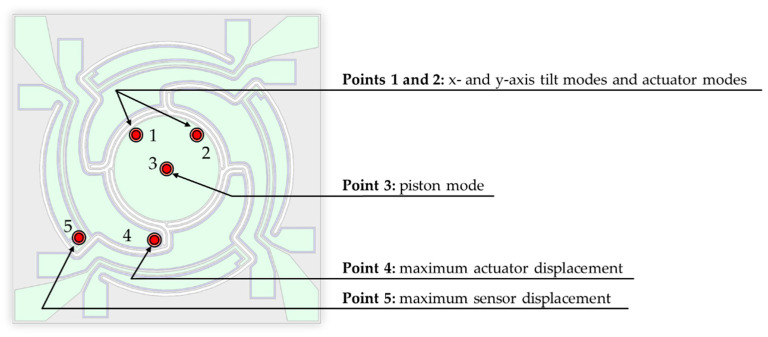
Measurement points for displacement determination.

**Figure 18:**
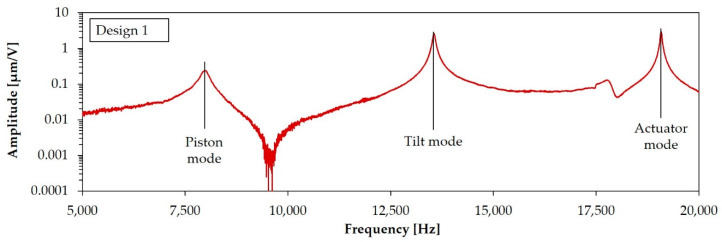
Frequency response curve of an individual sample of Design 1 (recorded at Point 1).

**Figure 19:**
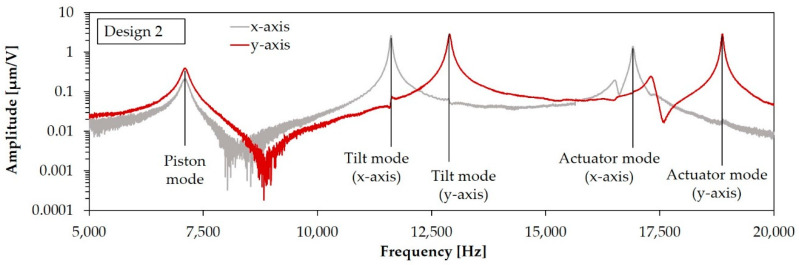
Frequency response curve of an individual sample of Design 2 for x- and y-axis (recorded at Point 1 and 2, respectively).

**Figure 20:**
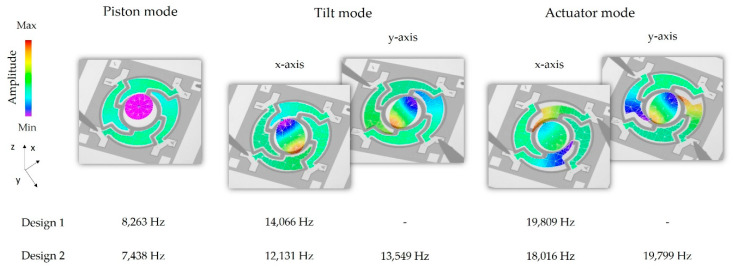
Out-of-plane modes, measured by laser-Doppler-vibrometry for Design 1 and Design 2.

This section shows the results of the optical measurements carried out with the LDV setups explained in Section 4.1. In Figure 17 the measurement spots of the displacement determination for the simulation and LDV measurement are exemplarily explained for Design 1-S. Figure 18 shows the frequency response curves for an exemplary sample of Design 1, recorded at measurement Point 1. In accordance with the simulation, a piston mode appeared first, which showed a characteristic z-displacement of the mirror plate. Then, the tilt mode appeared. The actuator mode appeared last. In this mode, the mirror plate and the actuators deflect out-of-phase to each other. A parasitic mode can be seen between the tilt and actuator modes. The frequency response curves for the x- and y-axis of Design 2 were recorded at measurements Point 1 and 2, respectively (see Figure 19). The same resonance modes appeared in the same order as for Design 1. However, the tilt and actuator modes were differentiated in the x-and y-axis. The tilt mode peaks for the x- and y-axis clearly differed from each other, which is necessary for an uncoupled movement in both axes. Figure 20 shows the corresponding motion scan images of the mentioned resonance modes for the individual samples. Table 4, Table 5 and Table 6 summarize the results of the LDV measurements (median values) over one wafer, in comparison to the adjusted FE simulation.

The values for the resonance frequencies measured in Figure 20 show a considerable deviation from the simulated target frequencies depicted in Figure 3, which can be attributed to the technological deviations mentioned in Section 2 and Section 3. After FE model adaptation, the eigenfrequencies simulated, including the technological deviations and intrinsic stresses, showed a mean deviation of ±2% from the measured mean resonance frequencies over the wafer. The simulated static deflections showed slightly larger deviations. Possible reasons are non-ideal layers by local defects or AlN thickness deviations, reducing the efficiency of the AlN actuators. In addition, the piezoelectric d_31_-coefficient might be lower than the assumed value of −2 pm/V. This uncertainty is a subject of further investigation.

### 5.3. Scanning Characteristics

**Figure 21:**
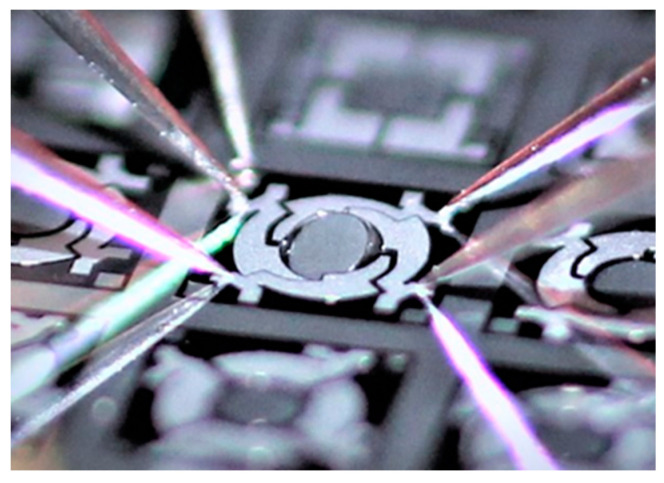
Photograph of a micromirror at wafer level of Design 2 in operation at 50 V (approx. 30° mechanical tilt angle) captured by a single lens reflex (SLR) camera (Canon EOS 600D).

**Figure 22:**
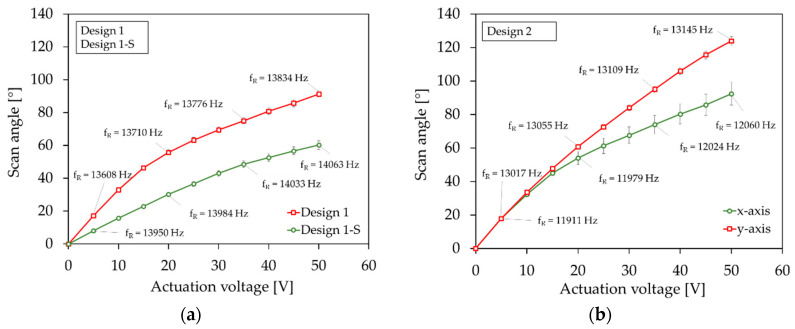
Resonant scan angle versus actuation voltage: (**a**) Design 1 and Design 1-S; (**b**) Design 2. The y-axis error bar is the median absolute deviation.

In this section, the scan properties of the micromirrors at high deflections for the tilt mode are identified with the high deflection setup explained in Section 4.2. The systems were operated with a sine wave signal, with voltages of up to 50 V. An example micromirror of Design 2 operating at a mechanical tilt angle of approximately 30° is shown in Figure 21. Figure 22 shows the resonant scan angle versus actuation voltage for Design 1 and Design 1-S (Figure 22a), and Design 2 (Figure 22b). The resonance frequencies *f_R_* were adapted to the scan angle accordingly. The curves show an average value that was calculated from three micromirror samples. An average resonant scan angle of 91.2° and 60.2° at 50 V was reached for Design 1 and Design 1-S, respectively. In comparison to Design 1 the scan angle of Design 1-S was smaller. This was due to the integrated sensor elements, which led to a reduction of the effective actuator area. For Design 2, an average resonant scan angle of 92.4° and 123.9° at 50 V was reached for the x- and y-axis, respectively. A quasistatic scan angle of 1.5° was measured at 100 V and 30 Hz for the y-axis. In general, Design 2 showed higher deflections than Design 1. A possible reason could be the loss of energy due to the unwanted, measurement principle dependent, excitation of the non-excited actuators, with an identical eigenfrequency, like the excited actuators of Design 1.

**Figure 23:**
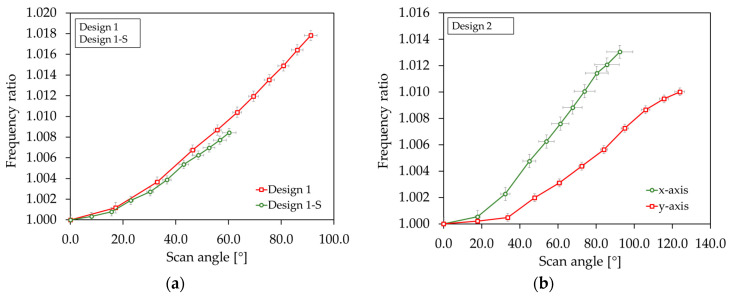
Frequency ratio versus scan angle: (**a**) Design 1 and Design 1-S; (**b**) Design 2. The x- and y-axis error bar is the median absolute deviation.

**Figure 24:**
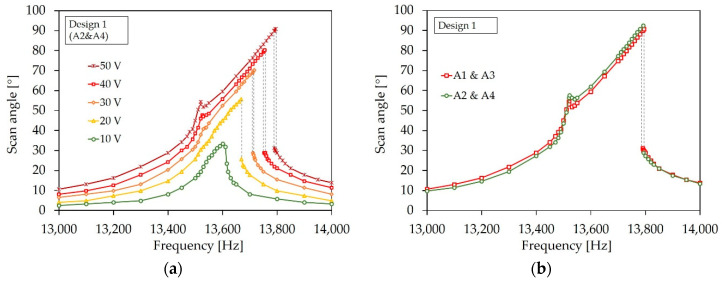
Frequency response curves of Design 1: (**a**) Actuation with actuator pair A2 and A4 for different voltages; (**b**) Comparison of the frequency response curves, generated at actuation of A1 and A3 and A2 and A4 at 50 V.

In Figure 23a,b, the frequency ratio versus scan angle is shown for the designs. The frequency ratio was determined by the ratio of the resonance frequency at the certain scan angle and the resonance frequency at small deflections of the corresponding sample device. The curves show an average value calculated from three example micromirrors. The frequency ratio increased for all designs significantly. The shift to higher frequencies indicates a non-linear deflection-dependent stiffening behavior. In Figure 24 the frequency response curves of a sample micromirror of Design 1 for different actuation voltages is depicted. Due to the measurement setup, only one pair of actuators could be excited to record a frequency curve. A non-linear behavior also became evident by a deflection-dependent shift of the resonance frequency to higher values. The frequency curves tilt towards higher frequencies. In addition, there is a hysteresis that extends with increasing scan angle (dashed lines). The hysteresis is expressed through different frequency curves for a forward or reverse frequency sweep. Depending on the sweep direction, the amplitude falls or rises suddenly if a certain frequency value is exceeded. Within the hysteresis range, the scanning angle jumps between the higher and lower state. In addition to the tilted main peak, a second peak appears in the frequency curve of Design 1 at a lower frequency, which also increases with the deflection (see Figure 24a). As can be seen in Figure 24b, the peak also arises when the second pair of actuators is excited independently. This indicates that the peak was caused by the other, non-excited pair of actuators, due to the almost identical resonance frequency.

**Figure 25:**
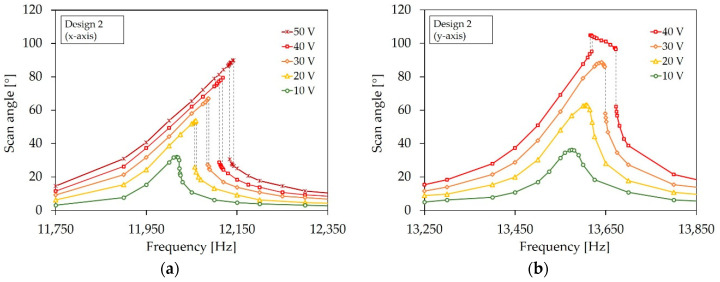
Frequency response curves of Design 2: (**a**) x-axis; (**b**) y-axis.

**Figure 26:**
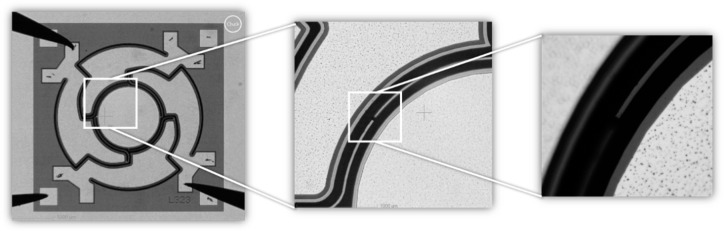
Microscope image of a micromirror of Design 2. After sweeping the frequency resonance curve at 50 V, one of the 7.5 µm springs was broken after an undefined oscillation occurred.

The frequency response curves for Design 2 in Figure 25a also reveal a non-linear stiffening behavior, due to a tilting of the curve towards higher frequencies and the occurrence of hysteresis. In contrast to Design 1, no second peak appears due to the different spring design for the x- and y-axis, leading to different resonance frequencies, and thus an uncoupled movement. However, in the frequency curve of the y-axis in Figure 25b mixed nonlinearities become evident. Up to 30 V, the frequency curve shows a typical stiffening behavior. At 40 V, a second peak appears near the main peak. The second peak has a hysteresis, and tilts to the lower frequencies, which indicates a softening behavior. Even though the scan curve could be recorded up to 50 V without destruction (Figure 22b), the micro systems were destroyed by sweeping through the frequency curve at this voltage. This phenomenon occurred regardless of the sweep direction. If the second peak was reached during sweeping, the laser line shows that the mirror did not perform a pure 1D movement at this point, and the micromirror broke at the thinner springs of the x-axis (see Figure 26). Mechanical stress probably arises, for example, from an undefined movement or the sudden increase in the amplitude of the microsystem, which leads to destruction at the weakest points.

Nonlinear effects occurring with MEMS have often been documented in the literature [1,26,27,28,29,30,31]. In general, nonlinear effects can be caused in terms of the spring constant or damping effects. Stiffening effects (progressive system behavior) are caused by a progressive spring design, or a reaction of the drive, in the form of a deflection-dependent restoring force. This results in an additional component in the total spring constant [1,27,28]. However, softening effects (degressive system behavior) are affected by nonlinear damping effects, e.g., due to temporally variable dimensions of fluid channels leading to a change of the fluidic damping component [1,31]. This can occur, for example, by the movement of the mirror plate relative to the actuators or to the fixed frame. In future work, the occurrence of nonlinear effects with regard to the MEMS design will be examined.

**Figure 27:**
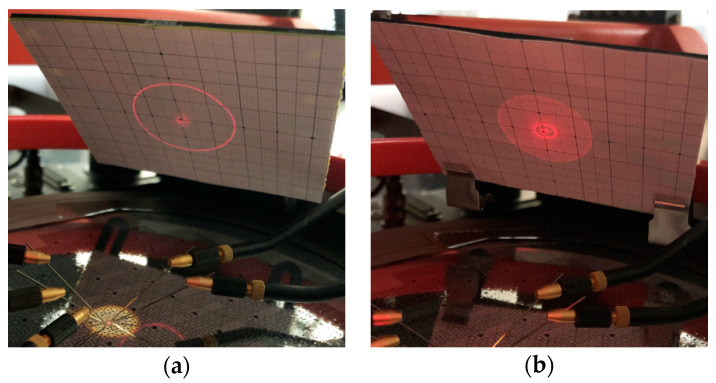
Scan pattern of Design 1 at 10 V: (**a**) spiral scan line without amplitude modulation; (**b**) spiral scan pattern by amplitude modulation.

**Figure 28:**
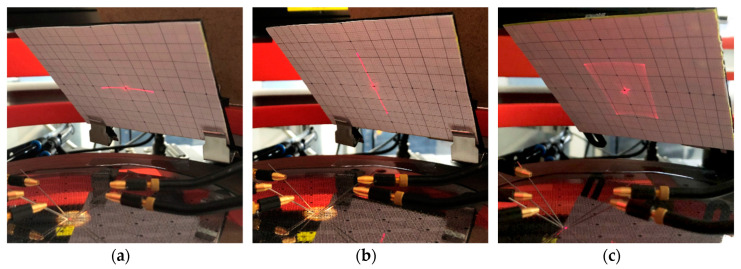
Scan pattern of Design 2 at 10 V: (**a**) x-axis; (**b**) y-axis; (**c**) Lissajous scan pattern.

In Figure 27 and Figure 28 the scan beams and scan patterns of Design 1 and Design 2 are depicted. The pattern generation was carried out according to Table 3 in Section 4.3, with an excitation voltage up to 10 V. A cross-shaped image appears in the center of both the scan lines and the scan patterns. This phenomenon occurs since the diameter of the laser beam is larger than the mirror plate itself and, thus, also illuminates the spring elements and actuators during operation. For Design 1 a uniform circle was generated at a drive frequency of 14 025 Hz (Figure 27a). Figure 27b shows a scan pattern generated with an amplitude modulation frequency of 25 Hz. A homogeneous scan area can be achieved, except for an unscanned area in the center of the circle. In future work, the scan pattern will be adjusted by optimizing the amplitude modulation function. In Figure 28a,b the 1D x- and y-scan lines of a sample micromirror of Design 2 are shown. A decoupled function of both axes is evident. A completed Lissajous scan pattern is shown in Figure 28c. The drive frequency for the x- and y-axis is 11,881 Hz and 13,497 Hz, respectively. Due to manufacturing tolerances in the measurement setup, the scan pattern was not projected as an ideal rectangle. Typically for Lissajous scans, the edge area is more exposed than the center. Nevertheless, a dense scan field is generated due to high drive frequencies. The resonance frequencies are adapted depending on the deflection. By making minor adjustments to the scan frequencies within the 3_dB_-bandwidth of the tilt mode, the scan pattern can be further optimized in terms of fill density and full-repetition imaging [15].

### 5.4. Sensor Characteristics

**Figure 29:**
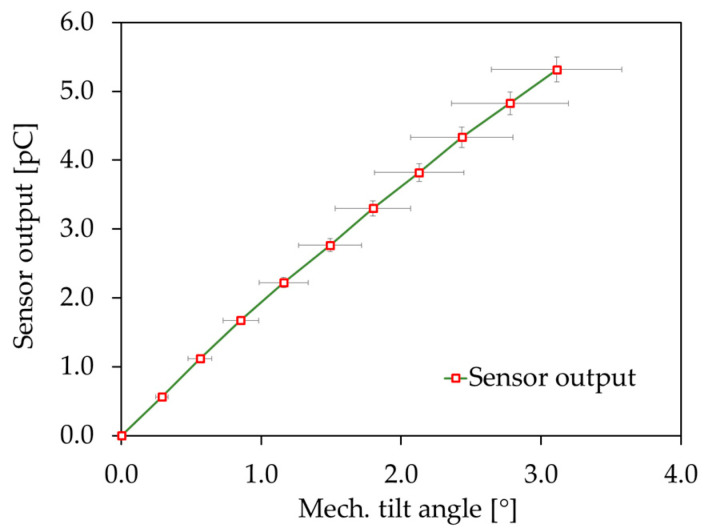
Sensor output charge versus mechanical tilt angle for Design 1-S in resonant operation (average of four sensor elements). The x- and y-axis error bars are the measured median absolute deviations.

In Figure 29 the sensor output charge versus the mechanical tilt angle is shown. The mechanical tilt angle was measured corresponding to Figure 17 at Point 1 and Point 2 in resonant operation, with the optical setup explained in Section 4.2. To determine the sensor angle sensitivity (defined as charge per mechanical tilt angle) the charge-equivalent sensor output voltage was recorded, with the sensor characterization setup explained in Section 4.4. Due to the I/O device, the micromirror sample was excited with a maximum voltage of 10 V, which resulted in a mechanical tilt angle of 3.1°. An average sensor angle sensitivity of 1.7 pC/° was calculated. To determine the sensor sensitivity (defined as charge per actuator deflection), the actuator deflection was measured at Point 5 by laser-Doppler-vibrometry. A mean sensor sensitivity of 0.5 pC/µm was reached.

## 6. Conclusions

Two-dimensional scanning micromirrors with large resonant scan angle were presented. The micro devices are based on piezoelectric AlN, which is used as a transducer material for actuator excitation and tilt angle detection. A possible application of the micromirrors is endoscopic in-vivo fluorescence microscopy, due to their small footprint. They can also be used for consumer electronics, e.g., for miniaturized laser projection systems.

Two micromirror designs with a small chip size of 4 mm^2^ were realized. Different spring parameters, to achieve a movement around the x- and y-axis, were chosen to generate spiral or Lissajous scan patterns. To enable a closed-loop position monitoring, and thus an accurate image formation and reconstruction, an additional layout with monolithically integrated AlN sensor elements was introduced. In order to achieve the targeted resonance frequencies, an iterative ANSYS FE simulation was carried out. The micromirror devices were successfully fabricated in 150 mm silicon-on-insulator (SOI) technology. Si (111) served as the device silicon layer to support a high C-axis growth of the AlN layer. By doping the Si (111), it was also used as bottom electrode for actuation. After fabrication, a further simulation was carried out to include technological deviations, such as underetching and intrinsic layer stress, to analyze their influences on the eigenfrequency. Although high intrinsic stresses were identified in the fabricated micromirrors, a minor influence on the eigenfrequencies in contrast to the technological deviations was observed. After adjusting the simulation, the results of the modal analysis fit, with a deviation of ± 2% to the results of the LDV measurements.

The fabricated micromirrors achieved high scan angles in air. With resonant excitation up to 50 V, a scan angle of 91.2° was reached for Design 1 at 13 834 Hz. For Design 2 a scan angle of 92.4° at 12,060 Hz, and 123.9° at 13,145 Hz, was reached for the x- and y-axis, respectively. 2D scans were performed successfully, and a sensor angle sensitivity of 1.7 pC/° was reached.

In future research, the spiral scan pattern of Design 1 is planned to be optimized by adjusting the amplitude modulation function. The occurrence of the nonlinearities identified for all designs will also be investigated. The curvature of the mirror plate is planned to be reduced by increasing the Si device thickness, or by depositing an additional aluminum layer on the backside of the mirror plate. In order to investigate the reliability of the microsystems, their survivability will be examined on statistically relevant micromirror samples, with regard to vibrations, shocks, and temperature changes. Furthermore, the implementation of AlScN is planned in future research to further increase the deflection due to its higher piezoelectric coefficients compared to AlN. According to the current state described in references [32,33], a five-times higher deflection at the same applied voltage is estimated for the presented micromirror designs. In Table 7 a comparison of resonant driven micromirrors based on piezoelectric AlN in the current literature and this work is given.

## Figures and Tables

**Table 1:** Overview of the designed spring widths in comparison to the resulting spring widths after fabrication. The target thickness of the device Si was 20 µm.

Design	Design 1	Design 1-S	Design 2	
			x-Axis	y-Axis
Scan pattern	Spiral	Spiral	Lissajous	
Designed spring width [µm]	10	10	7.5	10
Spring width after fabrication [µm]	8.5	8.5	6.0	8.5

**Table 2:** Parameter set for micromirror design and modeling [18,19,20,21,22,23].

Parameter	Axis	Designation	Unit	Si (111)	AlN	Al
Layer thickness	z	h_z_	µm	20.0	0.6	0.5
Young’s modulus	x	E_x_	GPa	168.9	308	68
	y	E_y_	GPa	168.9	308	68
	z	E_z_	GPa	168.9	308	68
Shear modulus	x, y	G_xy_	GPa	66.9	128.3	28.6
	y, z	G_yz_	GPa	57.8	128.3	28.6
	x, z	G_xz_	GPa	57.8	128.3	28.6
Poisson number	x, y	ν_xy_		0.262	0.2	0.32
	x, z	ν_xz_		0.182	0.2	0.32
	y, z	ν_yz_		0.182	0.2	0.32
Piezoelectric coefficient		d_31_	pm/V	-	−2	-

**Table 3:** Actuator excitation parameters to generate the corresponding scan patterns.

Actuator	Analog Output	Spiral	Lissajous	
			x-Axis	y-Axis
A1	AO0	*f_x_* = *f_y_*, φ = 0°	-	*f* = *f_y_*, *φ_y_* = 0°
A2	AO1	*f_x_* = *f_y_*, φ = 90°	*f* = *f_x_*, *φ_x_* = 0°	-
A3	AO2	*f_x_* = *f_y_*, φ = 180°	-	*f* = *f_y_*, *φ_y_* = 180°
A4	AO3	*f_x_* = *f_y_*, φ = 270°	*f* = *f_x_*, *φ_x_* = 180°	-

**Table 4:** Comparison of finite elements (FE) simulation and laser-Doppler-vibrometry (LDV) measurement of Design 1.

Parameters	Unit	Measurement Point	FE Simulation ^1^	LDV Measurement ^2^	Measurement Uncertainty
(Quasi)static deflections					
Mirror deflection	nm/V	1, 2	12	10.6	0.3
Actuator deflection	nm/V	4	34	29.4	0.7
	nm/V	5	25	20.8	1.3
Resonant deflections (tilt mode)					
Quality factor		1, 2	-	214	0.6
Mirror deflection	nm/V	1, 2	-	2361	189
Mech. Tilt angle	°/V	1, 2	-	0.39	0.03
Actuator deflection	nm/V	4	-	1499	259
	nm/V	5	-	1108	214
Resonance Frequencies					
Piston mode	Hz	3	8315	8263	175
Tilt mode	Hz	1, 2	14,139	14,066	265
Actuator mode	Hz	4	19,835	19,809	367

^1^ FE simulation: static and modal analysis, including intrinsic stresses and technological deviations (cumulative underetching: 1.5 µm). Device silicon thickness: 18.5 µm. Tolerances: ± 2%. ^2^ LDV measurements: quasistatic at 1500 Hz (mean value of 5 sample micromirrors) and resonant with 1 V chirp actuation (mean values of 69 sample micromirrors). Measurement uncertainty: mean deviation by measurements.

**Table 5:** Comparison of FE simulation ^1^ and LDV measurement of Design 1-S.

Parameters	Unit	Measurement Point	FE Simulation ^1^	LDV Measurement ^2^	Measurement Uncertainty
(Quasi)static deflections					
Mirror deflection	nm/V	1, 2	6.0	4.3	0.2
Actuator deflection	nm/V	4	17.1	14.8	0.7
	nm/V	5	12.3	8.5	0.5
Resonant parameters (tilt mode)					
Quality factor		1, 2	-	223	8.4
Mirror deflection	nm/V	1, 2	-	1012	40.9
Mech. Tilt angle	°/V	1, 2	-	0.17	0.01
Actuator deflection	nm/V	4	-	596	14.5
	nm/V	5	-	439	14.8

^1^ FE simulation: static and modal analysis, including intrinsic stresses and technological deviations (cumulative underetching: 1.5 µm). Device silicon thickness: 18.5 µm. Tolerances: ± 2%. ^2^ LDV measurements: quasistatic at 1500 Hz (mean value of 5 sample micromirrors) and resonant with 1 V chirp actuation (mean values of 69 sample micromirrors). Measurement uncertainty: mean deviation by measurements.

**Table 6:** Comparison of FE simulation and LDV measurement of Design 2.

Parameters	Unit	Measurement Point	FE Simulation ^1^		LDV Measurement ^2^		Measurement Uncertainty	
			x-axis	y-axis	x-axis	y-axis	x-axis	y-axis
(Quasi)static deflections								
Mirror deflection	nm/V	1, 2	9.2	14.2	6.9	13.9	0.3	0.4
Actuator deflection	nm/V	4	33	33	29.1	29.1	0.7	0.8
	nm/V	5	26	26	19.3	20.2	0.4	1.3
Resonant deflections (tilt mode)								
Quality factor		1, 2	-	-	281	219	10.2	7.8
Mirror deflection	nm/V	1, 2	-	-	2408	2642	126.2	98.1
Mech. Tilt angle	°/V	1, 2	-	-	0.39	0.43	0.02	0.02
Actuator deflection	nm/V	4	-	-	740	1287	182	61
	nm/V	5	-	-	460	949	87	55
Resonance Frequencies								
Piston mode	Hz	3	7491	-	7438	-	123	-
Tilt mode	Hz	1, 2	12,140	13,738	12,131	13,549	208	249
Actuator mode	Hz	4	18,261	20,043	18,016	19,799	111	379

^1^ FE simulation: static and modal analysis, including intrinsic stresses and technological deviations (cumulative underetching: 1.5 µm). Device silicon thickness: 18.5 µm. Tolerances: ± 2%. ^2^ LDV measurements: quasistatic at 1500 Hz (mean value of 5 sample micromirrors) and resonant with 1 V chirp actuation (mean values of 69 sample micromirrors). Measurement uncertainty: mean deviation by measurements.

**Table 7:** Comparison of resonant driven micromirrors based on piezoelectric AlN in the current literature and this work.

Specification	Unit	J. Shao [6]	K. Meinel [10]	This Work	
				Design 1-S	Design 2
Mirror plate area	mm^2^	0.04	0.64	0.5	0.5
Chip size	mm^2^	-	6	4	4
Drive frequency	kHz	63.3	3.4	14.1	12.1, 13.1
Drive voltage	V	5	20	50	50
Scan angle	°	4.0	137.9	60.2	92.4, 123.9
Sensor angle sensitivity	pC/°	-	0.05	1.7	-
Number of scan dimensions		2	1	2	2

## References

[1] Specht H. (2011). MEMS-Laser-Display-System: Analyse, Implementierung und Testverfahrenentwicklung. Ph.D. Thesis.

[2] Schenk H. (2000). Ein neuartiger Mikroaktor zur ein- und Zweidimensionalen Ablenkung von Licht. Ph.D. Thesis.

[3] Naono T., Fujii T. (2014). A large-scan-angle piezoelectric MEMS optical scanner actuated by a Nb-doped PZT thin film. J. Micromech. Microeng..

[4] Kobayashi T., Maeda R. (2007). Piezoelectric Optical Micro Scanner with Built-in Torsion Sensors. Jpn. J. Appl. Phys..

[5] Qian R., Wen Z. (2011). A Piezoelectrically Actuated Scaning Micromirror Integrated with Angle Sensors. Key Eng. Mater..

[6] Shao J., Li Q. (2018). AlN based piezoelectric micromirror. Opt. Lett..

[7] Stoeckel C. (2016). Piezoelektrische Aluminiumnitrid-Dünnschichten für Mikroelektromechanische Systeme. Ph.D. Thesis.

[8] Meinel K., Stoeckel C. Piezoelectric Scanning Micromirror with Large Scan Angle Based on Thin Film Aluminum Nitride. Proceedings of the 2019 20th International Conference on Solid-State Sensors, Actuators and Microsystems & Eurosensors XXXIII (TRANSDUCERS & EUROSENSORS XXXIII).

[9] Meinel K., Stoeckel C. Piezoelectric scanning micromirror with built-in sensors based on thin film aluminum nitride. Proceedings of the 2019 IEEE SENSORS.

[10] Meinel K., Stoeckel C. (2020). Piezoelectric scanning micromirror with built-in sensors based on thin film aluminum nitride. IEEE Sens. J..

[11] Pensala T., Kyynäräinen J. Wobbling Mode AlN-Piezo-MEMS Mirror Enabling 360-Degree Field of View LIDAR for Automotive Applications. Proceedings of the 2019 IEEE International Ultrasonics Symposium (IUS).

[12] Senger F., Albers J. (2020). A bi-axial vacuum-packaged piezoelectric MEMS mirror for smart headlights. MOEMS and Miniaturized Systems XIX, Proceedings of the International Society for Optics and Photonics, San Francisco, CA, USA, 1–6 February 2020.

[13] Gu-Stoppel S., Lisec T. (2019). A highly linear piezoelectric quasi-static MEMS mirror with mechanical tilt angles of larger than 10°. MOEMS and Miniaturized Systems XVIII, Proceedings of the International Society for Optics and Photonics, San Francisco, CA, USA, 2–4 February 2019.

[14] Hwang K., Seo Y. (2017). Microscanners for optical endomicroscopic applications. Micro Nano Syst. Lett..

[15] Hwang K., Seo Y.-H. (2017). Frequency selection rule for high definition and high frame rate Lissajous scanning. Sci. Rep..

[16] Bazaei A., Yong Y. (2012). High-speed Lissajous-scan atomic force microscopy: Scan pattern planning and control. Rev. Sci. Instrum..

[17] Gu-Stoppel S., Giese T. (2017). PZT-Actuated and -Sensed Resonant Micromirrors with Large Scan Angles Applying Mechanical Leverage Amplification for Biaxial Scanning. Micromachines.

[18] Fei C., Liu X. (2018). AlN piezoelectric thin films for energy harvesting and acoustic devices. Nano Energy.

[19] Kwak D., Kim J. Why Is (111) Silicon a Better Mechanical Material for MEMS: Torsion Case. Proceedings of the ASME 2003 International Mechanical Engineering Congress and Exposition. Microelectromechanical Systems.

[20] Gerlach G., Dotzel W. (2008). Introduction to Microsystem Technology—A Guide for Students.

[21] Tilli M., Motooka T. (2015). Handbook of Silicon Based MEMS Materials and Technologies.

[22] Zhang D., Wei B. (2017). Advanced Mechatronics and MEMS Devices II.

[23] Bhugra H., Piazza G. (2017). Piezoelectric MEMS Resonators.

[24] Stoeckel C., Kaufmann C. (2014). Pulsed DC magnetron sputtered piezoelectric thin film aluminum nitride—Technology and piezoelectric properties. J. Appl. Phys..

[25] Solonenko D., Schmidt C. (2019). The Limits of the Post-Growth Optimization of AlN Thin Films Grown on Si(111) via Magnetron Sputtering. Phys. Status Solidi B.

[26] Nabholz U., Schatz F. (2019). Spontaneous Parametric Down-Conversion Induced by Non-Degenerate Three-Wave Mixing in a Scanning MEMS Micro Mirror. Sci. Rep..

[27] Smits J., Fujimoto K. (2005). Microelectromechanical flexure PZT actuated optical scanner. J. Micromech. Microeng..

[28] Filhol F., Defa E. (2005). Resonant micro-mirror excited by a thin-film piezoelectric actuator for fast optical beam scanning. Sens. Actuators A Phys..

[29] Frangi A., Guerri A. (2017). Parametric Resonance in Electrostatically Actuated Micromirrors. IEEE Trans. Ind. Electron..

[30] Ataman C., Hakan U. (2004). Nonlinear frequency response of comb-driven microscanners. MOEMS Display and Imaging Systems II, Proceedings of the the International Society for Optics and Photonics, San Jose, CA, USA, 26–27 January 2004.

[31] Tsai Y. Metallic glass micro-mirror integrated with PZT actuation for low resonant frequency and large exciting angle. Proceedings of the 2013 Transducers & Eurosensors XXVII: The 17th International Conference on Solid-State Sensors, Actuators and Microsystems (TRANSDUCERS & EUROSENSORS XXVII).

[32] Akiyama M., Kamohara T. (2009). Enhancement of Piezoelectric Response in Scandium Aluminum Nitride Alloy Thin Films Prepared by Dual Reactive Cosputtering. Adv. Mater..

[33] Umeda K., Kawai H. Piezoelectric properties of ScAlN thin films for piezo-MEMS devices. Proceedings of the 2013 IEEE 26th International Conference on Micro Electro Mechanical Systems (MEMS).

